# A conceptual field-based framework for lower–upper body kinetic chain assessment in the tennis serve using IMUs and portable force plates: measurement coverage, new performance indices, and applied implications

**DOI:** 10.3389/fspor.2026.1784684

**Published:** 2026-04-07

**Authors:** Jun Woo Kwon

**Affiliations:** Department of Physical Education, Sports Technology Laboratory, Seoul National University, Seoul, Republic of Korea

**Keywords:** coordination, force plate, IMU, kinetic chain, tennis serve

## Abstract

Understanding the tennis serve requires an integrated view of how forces generated at the ground are transmitted through the lower limbs, trunk, and upper extremities to the racket. Despite this, most wearable-sensor research has focused predominantly on upper-body kinematics, with leg drive and ground reaction forces remaining largely unexplored in field-based settings. This conceptual paper proposes a practical, on-court framework that combines wearable inertial measurement units (IMUs) with a portable force plate to capture key lower–upper body components of the kinetic chain during the tennis serve. Building on established proximal-to-distal sequencing principles, two novel indices—the Kinetic Chain Efficiency Index and the Lower–Upper Coupling Ratio—are introduced to characterize lower–upper body coordination. In addition, a Measurement Coverage Index is presented to explicitly define which biomechanical components of the serve are accessible with this measurement configuration. By synthesizing existing literature and highlighting key methodological gaps, the paper outlines a conceptually grounded pathway for integrating lower-body mechanics into tennis biomechanics without reliance on laboratory-based motion capture. Practical implications for coaching and strength and conditioning are discussed, including potential applications in performance profiling, serve consistency, and the identification of kinetic-chain patterns that may be relevant to mechanical inefficiency or injury-related loading. Overall, the framework situates a more integrated biomechanical assessment within a realistic field environment and provides a structured basis for future research and applied tennis practice.

## 1 Introduction

The tennis serve is widely regarded as the most technically complex stroke in the sport, because it is the only self-initiated action and relies on a highly coordinated transfer of energy from the ground to the racket. The serve relies on what is commonly described as a kinetic chain, following a proximal-to-distal sequence in which ground reaction forces (GRFs), hip and knee extension, trunk rotation, and upper-limb acceleration are closely coordinated within a short time window (1, 2). When this sequencing is disrupted, serve velocity may decline, movement variability increases, and mechanical demands placed on the shoulder and elbow can increase, highlighting the central role of lower–upper body integration in both performance outcomes and injury-related loading (3).

While wearable IMUs have been applied in field-based biomechanics across a range of movement tasks, their application in tennis serve biomechanics has largely focused on upper-body kinematics. Many IMU-based serve studies emphasize trunk angular velocity, upper-arm rotation, and pelvis–trunk timing (4, 5), while giving less attention to the early and mechanically important elements of the chain, namely hip and knee extension, lower-body force production, and the initial GRF impulse. This omission is particularly consequential given the substantial contribution of leg drive to racket speed generation, its influence on trunk rotation timing, and its role in modulating upper-limb angular velocities (6, 7). When lower-body and GRF-related measures are not included, interpretations of serve mechanics may remain incomplete, particularly with respect to force transmission, movement consistency, and compensatory loading strategies that are relevant to coaching practice (8). In addition, lower-body IMU data remain largely underrepresented in the tennis biomechanics literature. Relatively few investigations have attempted to quantify hip or knee contributions, and these have mostly relied on laboratory-based optical motion capture rather than wearable systems (9, 10). Although optical motion-capture systems provide detailed kinematic information, they typically require specialized equipment, trained personnel, controlled laboratory conditions, and substantial setup time and cost, making them difficult to transfer to routine on-court assessment.

Although IMUs are reliable and valid for identifying various kinematic measurements within a tennis context, there is a lack of direct GRF measurement inclusion. In some movement tasks, force-related variables or GRF surrogates have been estimated indirectly from IMU-derived acceleration signals, particularly in walking, running, and jump-landing contexts, often using sensors placed near the trunk, shank, or foot (11–15). However, these approaches are typically developed for relatively cyclic or more mechanically constrained tasks, and their performance also depends heavily on modeling assumptions, signal-processing procedures, and sensor placement. By contrast, the tennis serve is a rapid, non-cyclic, whole-body movement involving complex lower–upper segment coordination and brief force-production events. Therefore, IMU-only estimation may not fully capture the magnitude and temporal characteristics of GRF during the serve. In this context, portable force plates offer a more direct basis for quantifying leg drive and its temporal relationship with trunk and upper-limb motion (2, 7). As a result, to the author's knowledge, few studies have examined lower–upper body kinetic-chain behavior in a genuine field environment through the combined use of lower-body IMUs and GRF data, leaving uncertainty about how energy is generated, transferred, and ultimately expressed during the serve (16). In addition to improving mechanical interpretation, combining IMUs with a portable force plate may also be advantageous in applied settings because it can provide lower-body force information in a format that remains feasible, interpretable, and deployable during routine on-court training.

The tennis serve represents a well suited movement for addressing this limitation due to its self-initiated structure, high reproducibility, and strong dependence on lower-body force production and trunk–arm sequencing, which together underpin the largest performance differences observed between elite and non-elite players (17, 18). In response to these gaps, this conceptual paper proposes a field-based framework that integrates wearable IMUs with a portable force plate to support a more integrated assessment of lower–upper body kinetic-chain components directly on court. Within this framework, two lower–upper body coordination metrics—the Kinetic Chain Efficiency Index (KCEI) and the Lower–Upper Coupling Ratio (CR)—are introduced alongside a Measurement Coverage Index (CI) that clearly indicates which biomechanical components of the serve are and are not accessible using this instrumentation. Through this structured and field-oriented approach, the present work aims to reduce the gap between laboratory biomechanics and applied coaching contexts by advancing a more integrated lower–upper body perspective on tennis-serve mechanics. Importantly, the present work should be interpreted as a conceptual framework rather than a fully validated measurement system. The proposed indices and measurement model are intended to provide a structured basis for future empirical investigation.

## 2 Literature review

### 2.1 Classic kinetic chain concepts

The kinetic chain refers to the coordinated interaction of body segments that generate, transfer, and amplify mechanical energy during dynamic movements. In tennis serving, this transfer follows a commonly reported proximal-to-distal sequence, beginning with forces applied at the ground and culminating in racket acceleration at ball contact (1, 2). Although this framework is straightforward in concept, the timing and coordination among segments must be well coordinated; small disruptions early in the sequence can have meaningful downstream effects (8).

A key element of chain-based performance is the stretch–shortening cycle (SSC), defined as the rapid transition from eccentric to concentric muscle action that enhances force production by preloading musculotendinous structures. During the serve, knee flexion before leg drive and trunk counter-rotation before rapid rotation illustrate SSC use, allowing the body to store and release elastic energy (6, 18). This mechanism is especially relevant for generating explosive segmental velocities with lower metabolic cost (1).

Another common theme within the literature is hip–shoulder separation, sometimes referred to as the X-factor. This angular offset between the pelvis and trunk provides a torsional stretch that primes the trunk for rapid rotation. Studies in baseball pitching, javelin throwing, and tennis often report positive relationships between greater hip–shoulder separation and increased distal velocities (8, 9, 19, 20). Without appropriate lower-body engagement, however, this separation is difficult to achieve (2).

Classic kinetic chain principles emphasize that the serve is not driven by the upper body alone; rather, it is the outcome of coordinated interactions among the legs, pelvis, trunk, and arm. This theoretical perspective highlights the value of measuring segments beyond the torso if the chain is to be understood in its entirety (21).

### 2.2 Current IMU-based tennis biomechanics research

Wearable IMUs are primarily used to quantify upper-body kinematics in tennis, particularly trunk and upper-arm rotational mechanics during the serve (4, 5, 22). In addition to research applications, IMUs have also been incorporated into commercial and on-court monitoring systems for tennis-specific load assessment (23). Tennis-specific investigations have demonstrated strong agreement between IMU-derived and optical motion capture measures of angular velocity, with correlations ranging from 0.951 to 0.993 and relative errors below 5% across stroke conditions (24). Previous studies have reported acceptable validity for IMU-based kinematic assessment relative to optical motion capture across upper-body segments and stroke-based movement tasks, including tennis-specific applications (25–27). However, the application of IMUs to lower-body mechanics in the tennis serve remains limited, with most studies focusing on upper-body sequencing or stroke-based analyses (5, 9, 10, 16). Nevertheless, IMUs have demonstrated acceptable to excellent validity and reliability in lower-body tasks such as gait and related movements, with reported correlations ranging from 0.71 to 0.99, sagittal-plane ICC values ranging from 0.60 to 0.99, and estimation errors as low as ∼4%–11% depending on the variable and method (14, 15, 28). These findings support the potential application of IMUs for evaluating lower-body contributions in the tennis serve context, although serve-specific validation remains needed.

However, IMU-based approaches do not directly measure GRF, which represents a critical mechanical input to the serve (2). In some movement tasks, force-related variables have been estimated indirectly from IMU-derived accelerations (11), particularly using sensors positioned near the shank or foot. These methods have demonstrated reasonable agreement with force-related measurements in selected activities. Although these approaches demonstrate the promise of wearable-only assessment, their transferability to the tennis serve remains uncertain because the serve involves rapid multi-segment coordination and a need to interpret lower-body force production relative to trunk and upper-limb motion. For this reason, direct GRF measurement via a portable force plate may offer an important methodological advantage when the goal is to characterize lower–upper kinetic-chain behavior more comprehensively (7, 11).

In sum, existing IMU-based studies provide useful but incomplete insight into serve mechanics. They illuminate trunk and upper-arm behavior but leave unanswered questions about how the movement is initiated and how lower-body actions contribute to overall performance (16).

### 2.3 Major research gaps

A review of the literature highlights several gaps that constrain current understanding of full-chain serve biomechanics: Table 1 summarizes these gaps identified in the current literature. These gaps point to the need for a unified framework capable of capturing key kinetic-chain components using portable, court-ready tools.

**Table 1:** Major research gaps in field-based tennis serve biomechanics.

Gap	Research gap	Description	Consequence for serve analysis
Gap 1	Absence of lower-body IMU data	Hip, knee, and ankle dynamics have rarely been measured using IMUs, despite their central role in initiating the kinetic chain (16).	The initiation phase of the serve cannot be characterized in field-based studies.
Gap 2	Limited validation of IMU-based GRF surrogates for the tennis serve	Although acceleration-derived estimates of force-related variables have been explored, their validity for representing GRF characteristics during the tennis serve remains insufficiently established relative to direct force-plate measurement (7, 11).	Lower-body force production and leg-drive magnitude cannot be reliably quantified.
Gap 3	No formal lower–upper chain coupling metric	Existing studies report intersegmental timing within the torso or upper limb but do not quantify interactions between lower-body extension and trunk or arm rotation (5, 24).	The mechanical and temporal integration of the kinetic chain remains unmeasured.
Gap 4	Absence of full-chain performance prediction models	Current serve prediction approaches rely on a limited set of kinematic variables and exclude the earliest phases of the kinetic chain (7, 9).	Performance models fail to account for the full force–motion pathway.
Gap 5	No integrated field-based framework combining IMUs and GRF	No existing framework integrates IMU and GRF data within a single, portable field-based system (16).	Coaches are left with tools that are either laboratory-dependent or insufficient for applied decision-making.

### 2.4 Practical demands from the coaching field

Coaches often emphasize the need for biomechanical feedback that is fast, interpretable, and applicable during regular training sessions. In practice, this means that measurements should be feasible on court, require minimal setup time, and produce insights that can be immediately translated into actionable techniques (29).

Raw IMU waveforms or uncontextualized angular velocities often provide limited value for coaching decisions. In practice, practitioners seek meaningful, integrated indicators—metrics that reflect the athlete's sequencing, power generation strategy, and movement efficiency (11, 22). Lower-body information is especially valued because many serve flaws originate at the legs rather than the arm, yet this information is rarely available outside the laboratory (30).

Portable force plates and wearable IMUs together represent a practical combination for capturing key lower–upper body dynamics in field settings. IMUs offer rich segment-level kinematics, while portable force plates provide the GRF data needed to contextualize lower-body drive (3). Notably, both technologies can be deployed courtside without compromising training flow, as suggested by recent validation studies of portable force platforms (31, 32).

The coaching field's demand for actionable, whole-body metrics has moved faster than current research tools. This provides a clear rationale for developing a new framework that unifies measurement, interpretation, and application (16).

## 3 Measurement coverage framework

### 3.1 Definition of the measurement coverage index (CI)

Assessing the kinetic chain of the tennis serve requires multiple biomechanical variables spanning kinematics, kinetics, and temporal coordination. However, not all of these elements can be captured using wearable IMUs or portable force plates. To address this, I propose the Measurement Coverage Index (CI)—a structured method for describing how comprehensively a given measurement system captures the components of a biomechanical construct (11).

The CI is defined as the proportion of required biomechanical variables that can be measured by IMUs or force plates during a field-based assessment. This approach acknowledges both the strengths and limitations of portable systems and provides a transparent foundation for interpreting full-chain analyses (11). The generic formula is:

$$\mathrm{CI}=\frac{\sum \left(\mathrm{directly}\;\mathrm{measurable}\;\mathrm{variables}\right)}{\sum \left(\mathrm{required}\;\mathrm{variables}\right)}$$

Although the CI can be expressed conceptually as either an unweighted or weighted proportion, the present paper uses the simplified formulation as a heuristic tool for illustrating measurement coverage in field-based systems. For example, trunk rotation velocity may be weighted more heavily than ball speed when analyzing serve mechanics because it is both more easily measurable and more strongly associated with sequencing efficiency (5). Any future weighting scheme should be grounded in empirical findings or expert-consensus approaches to maintain interpretability (29). The CI serves two purposes: (1) It clarifies the scope of what can be reasonably concluded from IMU and force-plate measurements; (2) It provides a reproducible benchmark that future researchers can adopt, critique, or refine, supporting more consistent reporting in field biomechanics evaluations (16).

In the present paper, the CI is intended as a conceptual classification aid rather than a validated quantitative score. To avoid overstating precision, the Coverage Index (CI), derived from the proportional definition above, is expressed on a four-level ordinal scale ranging from 0 to 3. This discrete scale is consistent with expert-rating approaches commonly used in applied biomechanics and technology validation, where measurement capability is often categorized rather than continuously quantified (33). The 0–3 structure also provides practical interpretability: a one-level increase corresponds to a practically noticeable improvement in measurable coverage, allowing researchers and coaches to compare systems without implying false numerical accuracy. In expert-rating approaches, categorical or ordinal judgment is often more realistic than using highly precise numerical values (29). For clarity, the CI is conceptually defined as a proportional representation of measurement completeness. To enhance practical interpretability and avoid overstating numerical precision, this proportional definition is subsequently expressed on a four-level ordinal scale (0–3). Accordingly, the CI values reported in this paper refer to the ordinal coverage level derived from the underlying proportional concept.

### 3.2 Essential biomechanical components of the tennis serve

To apply the CI, the fundamental components of serve biomechanics must be categorized according to the existing literature. I identify six categories commonly cited as essential for characterizing serve performance, variability, and efficiency; These components and their measurement feasibility using IMUs and a portable force plate are summarized in Table 2. This classification enables a structured evaluation of measurement completeness. By explicitly acknowledging which variables are accessible, the framework supports defensible interpretation of lower–upper chain patterns. The measurable components identified above are summarized in Table 3.

**Table 2:** Essential biomechanical components for characterizing tennis serve performance, variability, and efficiency.

Category	Biomechanical component	Key variables	Measurement modality	Notes
I	Trunk rotation dynamics (5, 22)	Angular velocity, rotational acceleration, timing of peak rotation	Measurable with torso IMU	—
II	Upper-arm angular velocity (24)	Internal rotation, elevation, and segmental speed	Measurable with upper-arm IMU	—
III	Hip and knee extension mechanics (11, 34)	Timing of extension, segment velocities, joint acceleration	Can be estimated using IMUs on the pelvis, thigh, and shank	Direct torques cannot be measured
IV	Ground reaction forces (GRF) and impulse (2, 7)	Vertical GRF, rate of force development (RFD), push-off timing	Measurable with portable force plate	—
V	Racket kinematics (racket-head speed) (5, 35)	Racket-head speed	Cannot be directly measured without a racket-mounted sensor	IMUs on the arm provide surrogates
VI	Ball outcome variables (ball speed, spin) (6, 7)	Ball speed, spin	Not captured with IMUs or force plates	Require radar or high-speed video

**Table 3:** Components of tennis serve biomechanics and their measurability.

Biomechanical component	Required in serve analysis	IMU measurable	Force plate measurable	Notes
Trunk rotational velocity	Yes	Yes	No	Key upper-body contributor of racket speed (5)
Upper-arm angular velocity	Yes	Yes	No	Relates to proximal-to-distal sequencing effectiveness (35)
Pelvis rotation	Yes	Yes	No	Captured via pelvis IMU orientation (24)
Hip extension	Yes	Yes	No	Estimated from thigh–pelvis IMU configuration (34)
Knee extension	Yes	Yes	No	Important component of leg drive (34)
Ground reaction force (GRF)	Yes	No	Yes	Fundamental measure of leg drive magnitude (2)
Vertical impulse	Yes	No	Yes	Integrates GRF over time (7)
Rate of force development (RFD)	Yes	No	Yes	Reflects explosive leg drive ability (7)
Racket-head speed	Yes	No	No	Not measurable with current equipment set (35)
Ball speed	Yes	No	No	Requires radar, camera, or ball-mounted sensor (36)

### 3.3 Example applications of the coverage Index

To demonstrate the interpretive value of the Coverage Index (CI), the framework can be conceptually applied to five representative constructs commonly analyzed in tennis serve biomechanics: serve power, serve consistency, leg-drive contribution, trunk rotation timing, and full kinetic-chain coordination. Each construct consists of a defined set of required biomechanical components, and the proportion of those variables directly measurable by IMUs and portable force plates is used here to illustrate a qualitative CI score ranging from 0 to 3.

These examples acknowledge that different performance constructs depend on different combinations of kinematic, kinetic, and temporal variables, many of which are only partially accessible in field-based measurement systems (16). By explicitly linking each construct to its measurable components, the CI helps prevent implicit assumptions that unmeasured variables are being captured or inferred reliably (11).

The CI is not intended to rank athlete performance, but rather to qualify the interpretive strength of biomechanical conclusions drawn from a given instrumentation setup. For instance, constructs such as trunk rotation timing can be assessed with relatively high coverage using IMUs (5), whereas leg-drive contribution and full kinetic-chain coordination require both GRF data and lower-body kinematics, resulting in lower CI values when force-plate data are absent (7). In this way, the proposed CI clarifies the measurement completeness of different performance domains while avoiding overinterpretation of variables that cannot be directly captured.

#### 3.3.1 Serve power

Serve power relies on the interaction between multiple mechanical sources, including trunk rotation, upper-arm angular velocity, pelvis rotation, hip and knee extension, and ground-reaction forces (GRF and impulse) (1, 2). Trunk and upper-arm rotational velocities represent the dominant internal contributors to racket acceleration near impact (5, 35), while hip and knee extension and GRF-related variables provide the proximal mechanical input that initiates and modulates this energy transfer (6, 7).

Although IMUs and a portable force plate can quantify the majority of these internal contributors, external performance outcomes such as racket-head speed and ball speed remain beyond the system's direct measurement capacity (35, 36). This domain is classified as moderate coverage (CI = 2), suggesting that most internal contributors to serve power are measurable, whereas the final output variables require additional instrumentation such as radar or optical tracking to be directly assessed.

#### 3.3.2 Serve consistency

Serve consistency is influenced by inter-trial variability in trunk rotation, the temporal stability of leg-drive timing, and the coordination between lower- and upper-body segments (3, 20). Variability in trunk and upper-limb kinematics, as well as fluctuations in GRF timing and impulse, has been shown to reflect differences in movement organization and control strategies across repeated serves (7, 20).

Wearable IMUs and portable force plates are capable of capturing most of these internal components, including kinematic variability and force–time characteristics of leg drive (17). However, outcome-level measures of consistency—such as ball placement accuracy, toss precision, or spin variability—cannot be assessed using the current sensor configuration and require additional sensing modalities (e.g., video or ball-tracking systems) (29). Therefore, serve consistency is rated as having moderate coverage (CI = 2): internal sequencing and force-production variability are observable, but full performance consistency cannot be quantified with IMUs and force plates alone.

#### 3.3.3 Leg-drive contribution

The leg-drive construct is determined by ground reaction force (GRF) magnitude, rate of force development (RFD), and the kinematics of hip and knee extension (2, 6). GRF-related variables, particularly vertical impulse and force–time characteristics, represent the primary mechanical input initiating the kinetic chain, while hip and knee extension kinematics describe how this force is generated and transmitted proximally (7, 34).

Several key components of leg-drive contribution are directly measurable using the combined configuration of wearable IMUs and a portable force plate. GRF magnitude, impulse, and RFD can be obtained from force-plate data, while hip and knee extension timing and segmental velocities can be estimated from pelvis, thigh, and shank IMUs without reliance on indirect surrogates (32). Consequently, leg-drive contribution is classified as high coverage (CI = 3), signifying that major field-measurable aspects of lower-body force generation can be characterized in a genuine field-based setting.

#### 3.3.4 Trunk rotation timing

Trunk rotation timing is primarily defined by trunk angular velocity profiles and pelvic orientation, including the timing of peak rotational velocity and pelvis–trunk phase relationships (5). Both variables are fully measurable through IMUs placed on the pelvis and trunk, which provide high-resolution temporal data of segmental rotations and have shown good validity and reliability in tennis serve analysis (4, 24).

Because rotational timing variables can be captured directly without reliance on surrogate measures or external instrumentation, trunk rotation timing receives a high coverage rating (CI = 3). This rating reflects that rotational-timing variables are among the most accurately and consistently measured quantities using wearable sensor systems in field-based tennis biomechanics (22).

#### 3.3.5 Lower–upper body coordination timing

Lower–upper body coordination in the serve involves the sequential timing of hip, pelvis, trunk, and arm segment peaks relative to the generation of ground reaction forces (GRF) (2, 9). Previous biomechanical studies have demonstrated that effective serve mechanics are characterized by consistent proximal-to-distal timing relationships, in which lower-body force production precedes pelvis and trunk rotation, followed by upper-limb acceleration (9, 37).

These temporal events can be directly derived from synchronized IMU and force-plate data streams, which allow precise identification of segmental peak angular velocities and GRF onset, peak, and impulse timing in field-based settings (24, 31). Although racket-head speed and ball outcome variables remain unmeasured within this configuration, they are not required for determining inter-segment timing relationships or coordination structure. As such, lower–upper body coordination timing receives a high coverage rating (CI = 3), reflecting that key temporal relationships can be evaluated using combined IMU and force-plate instrumentation (16).

Overall, the Coverage Index (CI) provides a structured means to evaluate how completely each serve construct can be quantified using field-deployable biomechanical tools. This approach improves transparency in reporting by distinguishing directly measurable variables from those requiring inference or supplemental instrumentation. The calculated Coverage Index (CI) scores for each major serve analysis domain are summarized in Table 4.

**Table 4:** Coverage Index (CI) scores for Major serve analysis domains.

Serve analysis domain	Required components	Measurable components	CI score (0–3)	Rationale
Serve power	Trunk rotation, upper-arm velocity, pelvis rotation, hip/knee extension, GRF/impulse, racket speed, ball speed	All except racket speed & ball speed	2 (Moderate Coverage)	Most internal contributors measurable; external outcomes not measurable (1, 2, 35, 36)
Serve consistency	Segment coordination, timing variability, trunk–pelvis pattern, GRF variability	All except ball outcome variability	2 (Moderate Coverage)	Movement variability measurable; outcome consistency not fully captured (3, 7, 20)
Leg-drive contribution	Hip/knee extension, GRF, impulse, RFD	All required measurable	3 (High Coverage)	Complete leg-drive profile measurable with IMU + force plate (2, 7, 31, 34)
Trunk rotation timing	Trunk angular velocity, pelvis orientation	Fully measurable	3 (High Coverage)	IMU accuracy high for rotational timing (4, 5, 24)
Full kinetic chain coordination	Hip–pelvis–trunk–arm sequencing, GRF timing	GRF and all joint timings measurable	3 (High Coverage)	Only racket/ball unavailable but not required for chain timing (9, 16, 37)

CI scores represent ordinal coverage levels derived from the proportional definition of the Measurement Coverage Index.

### 3.4 Importance of the coverage index for field biomechanics

The introduction of the CI addresses several longstanding challenges in wearable-sensor biomechanics. First, it provides transparency, allowing researchers to clearly articulate what aspects of the chain their system captures and what remains outside measurable bounds (11, 16). This transparency is particularly valuable in coaching settings, where practitioners must distinguish between directly measured values and inferred insights (29).

Second, the CI supports reproducibility. By offering a structured method for documenting measurement capacity, it enables future studies to compare frameworks, evaluate technological improvements, and align methodologies (16).

Third, the CI enhances practical interpretability. Coaches often rely on tools that provide limited or uneven information. By quantifying the breadth of biomechanical coverage, the CI helps practitioners understand how confidently they can interpret changes in serve mechanics and whether additional tools are required (22, 29).

Finally, the CI encourages the development of new performance metrics, such as the KCEI and Coupling Ratio presented in the next section. These indices explicitly rely on measurable components, making them conceptually suitable for future field-based application and evaluation (11).

## 4 Proposed lower–upper kinetic chain framework

### 4.1 Measurement system

The proposed field-based framework combines wearable inertial measurement units (IMUs) with a portable force plate to characterize selected features of the tennis serve sequence (16, 38). This combination provides a practical balance: IMUs provide continuous segment-level kinematics across the entire chain, while the force plate records the lower-body force profile that initiates upper-body motion (2).

For sensor placement, I suggest using Noraxon IMUs positioned on the ankle, knee, pelvis, trunk, and upper arm. This configuration covers the major contributors to chain sequencing. The ankle and knee sensors provide information about leg-drive mechanics and segment acceleration patterns (34, 39); the pelvis sensor captures hip rotation and serves as a bridge between lower and upper segments (24); the trunk and upper-arm sensors record the core rotational events that precede racket acceleration (4, 25).

**Figure 1:**
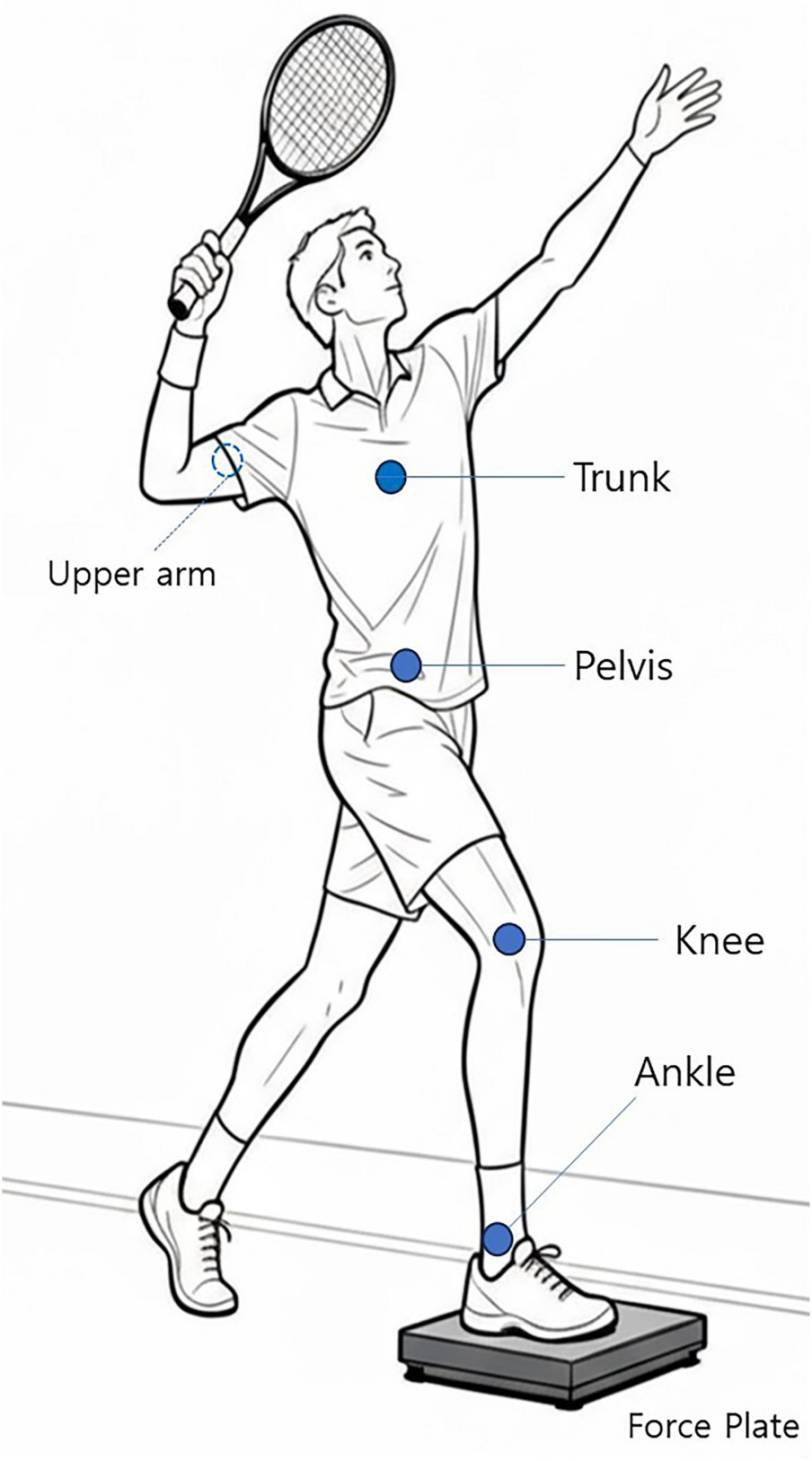
Measurement setup with IMU sensor attachment and force plate.

The Bertec portable force plate is proposed for measuring vertical GRF, impulse, and rate of force development (RFD) during serve-related lower-body force events, depending on placement and study objective (32, 40). The mobility of the device allows it to be placed courtside without interrupting natural serving mechanics. Synchronization between IMUs and the force plate can be performed by aligning time stamps or using a shared trigger signal, so that lower-body forces and segmental kinematics can be analyzed in relation to each other (41). Sensor placement and force plate are illustrated in Figure 1. This dual-system configuration provides a level of detail that may help bridge laboratory-derived measures and on-court assessment, allowing researchers and coaches to examine lower–upper body interactions during the serve (16, 17, 31, 32).

### 4.2 Proposed kinetic chain metrics

In this paper, three indices are introduced to characterize selected aspects of kinetic-chain behavior in a field-based environment: the Measurement Coverage Index (CI), the Kinetic Chain Efficiency Index (KCEI), and the Lower–Upper Coupling Ratio (CR). To my knowledge, these indices are not widely used in tennis biomechanics and are proposed here as conceptual metrics to describe lower–upper body coordination and measurement completeness in the tennis serve. These indices should be interpreted as conceptual constructs intended to operationalize specific temporal and mechanical relationships, rather than as fully validated performance metrics. To translate raw sensor signals into interpretable information, I introduce two novel indices designed to quantify the interaction between lower-body force generation and upper-body rotational dynamics (8, 16).

The tennis serve involves a proximal-to-distal kinetic chain in which force and motion are transferred sequentially across body segments over the course of the movement (1, 9, 42). The action is initiated at the ground, where ground reaction forces and lower-limb extension contribute to vertical and rotational impulse generation. Hip and knee extension precede pelvis rotation, providing the mechanical conditions required for effective trunk rotation. The trunk then plays a key intermediary role by transmitting energy from the lower body to the upper limb, after which shoulder, elbow, and wrist motions contribute to racket-head acceleration at ball contact (1, 9, 43).

Rather than consisting of discrete, independent actions, the serve reflects a continuous progression in which the contribution of each segment emerges slightly later than that of its proximal counterpart (42). When this temporal sequence is disrupted—for example, by insufficient leg drive or premature trunk rotation—the efficiency of energy transfer can be reduced, with potential consequences for both serve performance and loading of the upper extremity (9, 42, 43). This established conceptual understanding of kinetic chain sequencing forms the biomechanical basis for the lower–upper coordination metrics introduced in the present framework.

#### 4.2.1 Kinetic chain efficiency Index (KCEI)

The Kinetic Chain Efficiency Index (KCEI) is intended to capture the temporal relationship between lower-body extension and upper-body rotational acceleration (2, 9). Specifically, it evaluates how effectively the timing of hip and knee extension sets up the trunk-to-arm sequencing (19). The index is defined as:

$$\mathrm{KCEI}=\frac{t_{\mathrm{upper}\text{–}\mathrm{arm}\;\mathrm{peak}}-t_{\mathrm{trunk}\;\mathrm{peak}\;}}{t_{\mathrm{trunk}\;\mathrm{peak}}-t_{\mathrm{knee}\;\mathrm{extension}\;\mathrm{peak}}}$$

Lower KCEI values may indicate a tighter temporal coupling between segments, whereas higher values may reflect relatively greater delays, premature trunk rotation, or less efficient force transfer (3, 8). Importantly, this index can be derived using IMUs and the force plate, enabling field-based assessment of lower–upper body timing relationships (38).

At present, the KCEI should be interpreted primarily as a comparative conceptual metric; normative thresholds distinguishing “good” vs. “poor” values remain to be established empirically. In addition, future work may examine whether normalizing the index to the duration of the relevant serve phase improves comparability across athletes. For example, relatively smaller temporal spacing between lower-body and upper-body peak events may be interpreted as tighter sequencing, whereas larger spacing may indicate a looser or delayed sequence. These examples are illustrative only and should not be interpreted as normative thresholds.

#### 4.2.2 Lower–upper coupling ratio (CR)

While the KCEI captures timing relationships, the Lower–Upper Coupling Ratio (CR) is intended to reflect the mechanical interaction between trunk rotation and hip extension (8). In conceptual terms, it quantifies the ratio of trunk rotational acceleration to hip extension acceleration—an indicator of how effectively the lower body provides the mechanical foundation for upper-body motion (2, 8, 30, 37). In practical implementation, the CR would require explicit specification of whether peak or average acceleration values are used, the temporal window over which those values are calculated, and the filtering procedures applied to the IMU signals, given the sensitivity of acceleration-based measures to processing choices.

$$\mathrm{CR}=\frac{\mathrm{Trunk}\;\mathrm{Rotational}\;\mathrm{Acceleration}}{\mathrm{Hip}\;\mathrm{Extension}\;\mathrm{Acceleration}}$$

Lower CR values may reflect an underpowered or delayed hip drive, whereas excessively high values may indicate overreliance on upper-body rotation (30, 44, 45). The CR offers a complementary view of chain performance, balancing timing (KCEI) with mechanical contribution (46).

Together, the KCEI and CR provide complementary conceptual information on lower–upper body coordination by integrating temporal and mechanical components in a field-oriented format (26, 47, 48). Conceptually, a more balanced CR may reflect closer alignment between trunk rotational demand and hip-extension support, whereas markedly low or high values may suggest different forms of lower–upper body imbalance. At present, these interpretations remain illustrative and require empirical confirmation.

### 4.3 Rationale for index design

The KCEI and CR were developed in response to two limitations in the current literature. First, existing IMU-based studies focus almost exclusively on upper-body variables and therefore cannot characterize how lower-body forces shape trunk and arm actions (5, 22). Second, although GRF and hip/knee extension are recognized as critical contributors to serve performance, few frameworks have quantified how these variables interact with trunk rotation (2, 37, 40).

The proposed indices address both issues by grounding their structure in well-established biomechanical principles. The KCEI reflects the well-known proximal-to-distal timing sequence, capturing whether lower-body extension precedes trunk rotation and whether trunk rotation appropriately sets up the arm segment (8, 9). The CR is rooted in mechanical reasoning: trunk rotation is not an isolated action but is influenced by the magnitude and timing of hip extension forces, which determine the potential for rotational acceleration (19, 30).

One advantage of these metrics is their potential measurability in field environments. Several required components—such as segmental peaks, angular accelerations, and lower-body force characteristics—can be captured using IMUs and a portable force plate (24, 26, 31). This stands in contrast to laboratory-derived metrics such as joint torques or inverse-dynamics estimates, which require motion-capture arrays and cannot be replicated courtside (49, 50).

By translating complex biomechanical interactions into interpretable indices, this framework may help inform coaching decisions and future research on performance, injury-related loading, and long-term athlete development (16, 44, 46).

## 5 Conceptual on-court implementation

This section outlines how the proposed framework can be implemented on court, showing how full-chain analysis could be performed without laboratory infrastructure (26, 38, 51). The procedures are presented as a repeatable, coach-friendly protocol that maintains biomechanical rigor while remaining feasible in typical training environments (16, 22, 31).

### 5.1 On-court measurement protocol

To evaluate practical feasibility conceptually, the system is designed for on-court deployment on a regulation tennis court, with the measurement process structured to minimize interference with natural serving mechanics (16, 22, 26). In a typical implementation, athletes would complete a standardized warm-up that includes dynamic stretching and several sub-maximal serves to ensure consistent neuromuscular readiness (47). IMUs would then be placed on the ankle, knee, pelvis, trunk, and upper arm using adhesive mounts and elastic straps to reduce soft-tissue movement (4, 25). Each sensor would be initialized in a neutral standing posture to establish a common reference orientation (24).

One possible implementation would position the portable force plate at the athlete's lead-foot landing location during the serve (31, 40). This placement is presented as a practical field-based option rather than a definitive solution for all biomechanical objectives, and alternative placements may be required depending on whether the emphasis is on landing-related force events, push-off characteristics, or broader inverse-dynamics considerations. Because serve footwork is relatively consistent across trials, athletes would usually require only a brief familiarization period to comfortably land on the plate without altering their technique (17). The force plate should remain flush with the court surface to avoid altering stance height or stability (32).

Following this setup, athletes could perform a set of serves at match-representative intensity. To keep the setting representative, balls would not be constrained and no instructions regarding target location would necessarily be imposed (16). All measurements could be recorded in real time, mirroring the demands of applied coaching environments (22). This protocol is intended to illustrate how a dual-system setup could be executed with limited burden on athletes while remaining compatible with field biomechanics applications (11, 38).

### 5.2 Data synchronization and processing

Because the IMU system and the force plate typically operate independently, synchronization is required for deriving chain-level metrics (41). One practical approach is to use a two-step alignment method in which (1) both systems are time-stamped using a shared computer clock, and (2) a characteristic “initial loading spike” in the vertical GRF is matched with the onset of ankle acceleration to refine the alignment (31). As an alternative, a simple physical tap event between an IMU unit and the force plate before data collection may also provide a clear synchronization spike in both data streams.

#### 5.2.1 IMU processing

IMU data can be processed using a fusion algorithm (e.g., Kalman filter or proprietary AHRS filtering) to generate segment orientation, angular velocity, and angular acceleration (4, 24, 25). For each segment, the following features are of primary interest:

1. Peak knee and hip extension acceleration
2. Peak trunk rotational velocity and acceleration
3. Upper-arm peak angular velocity

Peak events can be identified using a low-pass Butterworth filter within an appropriate cutoff range (e.g., 20–40 Hz), selected to balance noise reduction and signal fidelity (48). For the KCEI, event timing should be defined explicitly with reference to the selected segmental peaks, whereas for the CR, the use of peak vs. average acceleration values and the corresponding analysis window should be specified *a priori*.

#### 5.2.2 Force plate processing

Vertical GRF can be filtered using a 25 Hz low-pass filter (49, 50). From these data, the following variables can be computed:

1. Vertical impulse during the preparation and drive phases
2. Rate of force development (RFD) during push-off
3. Peak vertical GRF as an indicator of leg-drive magnitude

Force data are typically normalized to body weight to enable meaningful comparisons across athletes (31, 40).

#### 5.2.3 Integration of IMU and GRF data

Once the two data streams are aligned, lower–upper body coordination patterns can be examined by evaluating:

1. Temporal offsets between knee extension peaks and trunk rotation peaks (2, 9)
2. Relationships between GRF impulse and trunk/arm accelerations (7, 37)
3. Mechanical coupling patterns used for CR calculation (8, 30)

This integration allows, in principle, an evaluation of how effectively an athlete converts lower-body force into upper-body rotational motion—something that IMU-only approaches cannot fully address (5, 16, 22).

### 5.3 Sources of error and mitigation strategies

Field biomechanics often involve noise and variability. However, most issues can be controlled through careful protocols and appropriate filtering (11). The main sources of error that need to be considered in the proposed framework, along with potential mitigation strategies, are summarized below.

1. IMU drift

Long recording sessions may lead to orientation drift (38).

Mitigation:

- Limit trial windows (e.g., 5–10 serves per session),
- use sensor fusion algorithms with magnetometer correction (4, 25), and
- periodically re-initialize sensors in a standardized T-pose (24).

(1) Soft-tissue artefact

Movement of sensors relative to the skin can distort peak detection (11).

Mitigation:

- Combine elastic wraps with adhesive interfaces,
- place sensors on bony landmarks where possible (26), and
- exclude anomalous spikes using median-based filters (48).

(1) Force plate placement and foot targeting

Athletes may miss the force plate if it is positioned too narrowly (40).

Mitigation:

- Position the plate under the lead foot’s typical landing zone (17),
- allow familiarization trials to ensure comfort (31), and
- use court tape markers to guide the approach stance when necessary.

(1) Synchronization errors

Small misalignments can distort timing-based indices such as KCEI (3).

Mitigation:

- Combine clock-based and GRF/IMU event-based synchronization (41),
- use cross-correlation to refine alignment (41), and
- conduct sensitivity analyses to verify the robustness of peak timing (49, 50).

(1) Filtering artefacts

Over-filtering can bias peak values, whereas under-filtering increases noise (48–50).

Mitigation:

- Apply cutoff frequencies matched to the angular velocities typical of the tennis serve (9), and
- verify consistency of results across low-, medium-, and high-intensity serves (47).

Through these procedures, the system is intended to yield stable and interpretable outputs suitable for both research-oriented and applied coaching environments, without requiring laboratory infrastructure (16).

## 6 Applied implications & research opportunities

The proposed framework extends beyond methodological innovation and offers practical insights that can influence coaching, player development, and sports-science research (18, 29). By integrating lower- and upper-body measurements in a portable, court-based environment, the framework may support future applications related to performance enhancement, biomechanical screening, and individualized technical intervention (8, 44, 46). This section outlines applied implications as well as avenues for future empirical studies (16).

### 6.1 Serve performance prediction

The two primary indices introduced in this paper—the Kinetic Chain Efficiency Index (KCEI) and the Lower–Upper Coupling Ratio (CR)—provide a compact representation of how effectively an athlete links force production and rotational sequencing (8). These indices may provide candidate inputs for future predictive models of performance outcomes such as ball speed, serve consistency, and point-to-point variability (7, 16).

#### 6.1.1 KCEI as a predictor of ball speed

KCEI reflects the temporal spacing between peak knee extension, trunk rotation, and upper-arm acceleration (9). Athletes who demonstrate a narrow, well-organized timing sequence typically generate faster racket-head speeds and, consequently, higher ball velocities (2, 3). Although the proposed framework does not directly measure ball speed, the underlying mechanics that govern velocity are captured with high sensitivity (18). This suggests a possible avenue for future surrogate ball-speed modeling using machine learning or regression techniques, pending empirical validation (5).

#### 6.1.2 CR as a predictor of consistency

Serve consistency relies on repeatable kinetic sequencing rather than maximum force production (20). The CR metric quantifies how effectively trunk rotational acceleration is supported by hip extension acceleration (37). Conceptually, players with a more stable CR across trials may be more likely to reproduce similar serve trajectories (17). This provides coaches with an actionable indicator of whether inconsistencies arise from mechanical breakdowns in the chain or from coordination issues elsewhere (29). Together, KCEI and CR can form the foundation for a new class of predictive tools tailored to field conditions—something currently absent in tennis biomechanics literature (5, 16).

### 6.2 Coaching applications

A central aim of this conceptual framework is to create metrics that coaches can interpret without requiring specialized biomechanical training (18, 29). The proposed system converts raw sensor data into decision-ready information (22).

#### 6.2.1 Elite vs. amateur classification

Coaches often struggle to articulate the mechanical differences between developing and advanced players. Future empirical work may determine whether KCEI ranges can help identify whether an athlete’s sequencing aligns with patterns observed in high-performance servers (9, 37). Similarly, CR stability can distinguish players who rely excessively on upper-body rotation from those who generate power efficiently through leg drive (8, 30).

#### 6.2.2 Real-time or near–real-time feedback

Because IMUs and portable force plates provide rapid processing, the system can support near-immediate feedback after small sets of serves (26, 31). Coaches can highlight whether an athlete is:

- initiating trunk rotation prematurely (3),
- underutilizing leg drive at push-off (7, 40), or
- generating inconsistent timing between lower- and upper-body peaks (20).

Even without laboratory-grade precision, such feedback holds substantial value during training sessions where iterative corrections are central to motor learning (29).

#### 6.2.3 Individualized technical adjustments

If supported by future empirical work, the framework could facilitate more personalized intervention. For example:

1. An athlete with strong GRF but a low CR may require rotational control drills (45, 46).
2. An athlete with high CR but a poor KCEI may need to improve knee/hip extension timing (19, 34).
3. Large trial-to-trial fluctuations in either metric can indicate neuromuscular fatigue, prompting training load adjustments (47).

This aligns the framework directly with applied coaching priorities-improving movement quality, maximizing efficiency, and reducing mechanical “energy leaks” (8, 44).

### 6.3 Injury-related mechanical considerations

Although the present paper does not aim to construct an injury-prediction algorithm, the framework is conceptually consistent with known mechanical factors associated with injury risk in tennis (8, 44). Poor kinetic chain coordination is a documented contributor to shoulder and elbow overload, especially during high-velocity serves (3, 30).

#### 6.3.1 Early trunk rotation

IMU-derived trunk angular acceleration allows for detection of premature trunk rotation, which can shift the mechanical burden onto the shoulder and elbow (3, 45). Coaches can quantify how early or late trunk rotation occurs relative to lower-body extension—a capability not previously available in portable systems (5, 24).

#### 6.3.2 Hip–shoulder separation breakdown

Limited separation between hip and trunk rotation reduces the ability to store elastic energy (9, 19). This deficiency often leads athletes to compensate using the arm alone, increasing the risk of upper-limb overuse injuries (44). The CR metric highlights precisely these imbalances by comparing trunk rotational demand with hip extension support (37, 46).

#### 6.3.3 Overreliance on upper-body rotation

Players exhibiting high upper-arm acceleration but weak lower-body force may fall into an injury-prone pattern (2, 30, 37). The combination of force plate data and IMU kinematics may help identify these discrepancies for further monitoring and intervention planning (26, 40).

In sum, the framework offers a pathway toward integrating performance and injury-prevention perspectives within the same measurement model—a long-standing need in applied tennis science (8, 16, 44).

### 6.4 Extension to other strokes

Although the tennis serve is the most suitable movement for introducing a lower–upper kinetic chain framework—given its reproducibility, discrete phases, and heavy reliance on GRF—the conceptual approach is not limited to serving (1, 16). With limited adaptation, the same IMU–force plate integration can be extended to other stroke types (24, 38).

#### 6.4.1 Forehand and backhand

Groundstrokes rely heavily on trunk–pelvis rotation and lower-body weight transfer (22, 26). By capturing foot–ground interaction and segmental timing, the framework can delineate technical inefficiencies that cannot be seen through video alone (29).

#### 6.4.2 Return of serve and volley

Fast-reaction strokes involve shorter preparation windows and smaller chains, yet the measurement principles remain applicable (42). Temporal sequencing indices may help identify the degree to which athletes maintain chain efficiency under time pressure (20).

#### 6.4.3 Movement transitions

Footwork patterns such as open-stance hitting, dynamic braking, or lateral acceleration can be incorporated into future iterations of the Coverage Index, particularly when evaluating load management in training sessions (11, 47).

Future research could evaluate how chain metrics evolve across stroke types, competitive levels, fatigue states, and even playing surfaces (16, 39). This expansion underscores the long-term versatility of the proposed framework (8).

## 7 Discussion

This paper presents a conceptual field-based framework for assessing lower–upper body kinetic-chain behavior in the tennis serve using a practical combination of IMUs and a portable force plate (16, 38). The framework questions the long-standing emphasis of laboratory-only kinetic chain assessment and addresses critical gaps in the existing IMU-based literature, particularly the near-total absence of lower-body measurements (5, 22). This section synthesizes the proposed contributions, contextualizes them within prior work, and highlights the broader significance for applied tennis biomechanics and coaching practice (8, 18, 29).

### 7.1 How this framework fills the research gaps

Previous research has consistently emphasized the importance of the kinetic chain in generating racket speed and maintaining serve consistency (1, 2, 9). Yet most IMU-based studies have examined only a subset of this chain—primarily trunk and upper-limb rotations—leaving the role of the lower body largely unmeasured (5, 16, 22). The present framework addresses this gap by explicitly integrating hip and knee kinematics with GRF-derived measures of leg drive (7, 37, 40).

By capturing (a) the temporal organization of lower-body extension, (b) trunk rotational behavior, and (c) upper-arm angular velocity peaks, the framework aims to characterize key elements of the segmental sequence underlying serve mechanics (8, 9). Importantly, this integration is achieved with equipment that can be deployed directly on court, not solely in controlled laboratory settings (24, 26, 38). From a methodological standpoint, the inclusion of the Measurement Coverage Index further clarifies what each sensor modality can and cannot capture, thereby improving transparency and reproducibility—two issues that have historically limited applied biomechanics studies (16, 17).

### 7.2 IMU-only vs. IMU + force plate: a methodological leap

A key contribution of this paper is to argue that adding a portable force plate may substantially expand the interpretive value of IMU data (31, 32). IMU-only systems can track rotational speeds, tilt angles, and limited sequencing variables, but they provide no information about the magnitude or quality of lower-body force production (5, 22). This omission is non-trivial: GRF characteristics such as impulse and rate of force development shape the entire upstream chain (2, 7, 37).

In contrast, combining IMUs with a portable force plate may provide a more informative basis for interpreting not only how segments move, but also how lower-body force events relate to those movements (8). Trunk rotational acceleration can now be interpreted in relation to hip extension acceleration and GRF impulse (9, 19). This coupling enables coaches and researchers to distinguish between athletes who rotate rapidly because they have generated substantial force at ground contact vs. those who rotate rapidly due to compensatory upper-body mechanics (30, 52). Such distinctions are crucial for evaluating performance potential, identifying mechanical inefficiencies, and preventing injury (44, 46).

This shift—from surface-level kinematics to integrated force–motion interpretation—extends what can be interpreted in on-court biomechanics (11, 16).

### 7.3 Why lower-body inclusion changes interpretation

The lower body plays a decisive role in determining both the magnitude and the temporal structure of kinetic chain transfer (2, 9, 37). Without lower-body data, interpretations of serve mechanics remain incomplete and, in some cases, misleading (5, 22). For example, an athlete may demonstrate high trunk rotational velocity but still produce a relatively slow serve if that rotation is not supported by adequate leg drive (7, 30). Conversely, large GRF at push-off may fail to translate into racket speed if timing mismatches exist between hip extension and trunk rotation (3, 19).

By attempting to quantify these lower-body contributions, the framework may support a deeper interpretation of serve performance (8). KCEI captures the coherence of the kinetic sequence, while CR reflects how effectively lower-body force production supports trunk rotation (9, 52). These metrics reveal where and when kinetic inefficiencies occur—a level of precision not possible with IMU-only systems (5, 16).

For coaching applications, this refinement is especially valuable. It enables practitioners to identify whether mechanical problems originate in force generation, segmental timing, or compensatory trunk or arm strategies, thereby directing training interventions to the appropriate location in the chain (18, 29, 44).

### 7.4 Value for federations, academies, and S&C coaches

The practical value of this framework extends to organizations seeking scalable, reliable tools for performance monitoring (18, 29). National federations and high-performance academies often require biomechanical insights but lack access to laboratory facilities (16). Similarly, strength and conditioning coaches need field-ready metrics that can guide load management, detect fatigue-induced mechanical drift, and track the long-term development of kinetic sequencing (44, 47).

The portability and ease of use of the proposed system support precisely these needs (31, 38). Coaches may gain access to interpretable outputs such as KCEI, CR, and Coverage Index values, which could help inform decisions about technical priorities, conditioning loads, and injury-prevention strategies (8, 46). Beyond individual athletes, federations can use these metrics as part of talent identification programs or longitudinal monitoring initiatives, thereby embedding biomechanics into high-performance pipelines without excessive cost (18, 53).

In summary, the proposed framework delivers scientific rigor while remaining practical and coach-friendly—a combination not always achieved in tennis biomechanics research (16, 29). Several limitations of the present work should be acknowledged. First, the framework is conceptual and does not constitute a full experimental validation of the proposed measurement model or indices. Second, the proposed CI, KCEI, and CR should be interpreted as heuristic constructs intended to guide future empirical work rather than as definitive performance metrics. Third, practical implementation details—including force-plate placement, synchronization strategy, signal filtering, and the definition of acceleration-based variables—may influence the resulting values and therefore require explicit specification in future validation studies.

## 8 Conclusion

This conceptual paper presented an on-court framework for evaluating the tennis serve through a more integrated lower–upper body kinetic-chain perspective using wearable IMUs and a portable force plate. The approach addresses common limitations in the existing literature, particularly the omission of lower-body mechanics and the absence of practical methods for capturing ground-based force contributions outside laboratory environments. By bringing together segmental kinematics and GRF-derived variables in a deployable, court-side configuration, the framework connects high-level biomechanical theory with the realities of applied coaching practice.

Two new metrics-the Kinetic Chain Efficiency Index (KCEI) and the Lower–Upper Coupling Ratio (CR)- were proposed to characterize the temporal and mechanical coherence of selected aspects of the serve’s kinetic sequence. These indices, grounded in classical proximal-to-distal principles, offer interpretable measures that can highlight whether an athlete's performance is driven by effective force transmission through the chain or whether compensatory strategies mask underlying inefficiencies. Together with the Measurement Coverage Index, which clarifies what components of serve biomechanics can be directly measured with field-ready tools, the framework enhances transparency and reproducibility while offering a structured basis for future empirical investigations.

More broadly, the work underscores the importance of incorporating lower-body dynamics into the evaluation of serve performance, a dimension that has been largely neglected despite its central role in generating and regulating racket speed. By outlining how IMUs and a portable force plate may jointly capture key mechanical signatures of leg drive, trunk acceleration, and upper-limb sequencing, this paper outlines a practical path toward improving field biomechanics beyond descriptive motion capture toward integrated force–motion interpretation.

Looking ahead, the framework creates a basis for future empirical validation and may support later work on machine-learning–based performance prediction and injury-related modeling. It also offers federations, academies, and strength and conditioning practitioners a scalable and interpretable system capable of supporting talent development and long-term athlete monitoring. In practical terms, by situating kinetic chain analysis where it matters most—in the athlete's actual performance environment—the approach presented here provides a foundation for more targeted, court-relevant investigation into a central high-impact skill in tennis.
