# Correction: A conceptual field-based framework for lower–upper body kinetic chain assessment in the tennis serve using IMUs and portable force plates: measurement coverage, new performance indices, and applied implications

**DOI:** 10.3389/fspor.2026.1851470

**Published:** 2026-04-22

**Authors:** Jun Woo Kwon

**Affiliations:** Department of Physical Education, Sports Technology Laboratory, Seoul National University, Seoul, Republic of Korea

**Keywords:** coordination, force plate, IMU, kinetic chain, tennis serve

In the Generative AI Statement, it was erroneously omitted that generative AI tools were used in the preparation of Figure 1. The correct statement is:

“The author(s) declared that generative AI was used in the creation of this manuscript. The author used generative artificial intelligence tools for checking and improving grammar and spelling, and for the preparation of Figure 1. No generative AI tools were used to generate scientific content, analyze data, interpret results, or formulate conclusions. The author takes full responsibility for the content of the manuscript.

Any alternative text (alt text) provided alongside figures in this article has been generated by Frontiers with the support of artificial intelligence and reasonable efforts have been made to ensure accuracy, including review by the authors wherever possible. If you identify any issues, please contact us.”

The original version of this article has been updated.

